# Myeloid-derived suppressor cells and pulmonary hypertension

**DOI:** 10.3389/fimmu.2023.1189195

**Published:** 2023-06-07

**Authors:** Hui Zhang, Qi-Wei Li, Yuan-Yuan Li, Xue Tang, Ling Gu, Han-Min Liu

**Affiliations:** ^1^ Key Laboratory of Birth Defects and Related Diseases of Women and Children (Sichuan University), Ministry of Education, West China Second University Hospital, Sichuan University, Chengdu, China; ^2^ The Fifth People’s Hospital of Chengdu, Chengdu, China; ^3^ Sichuan Birth Defects Clinical Research Center, West China Second University Hospital, Sichuan University, Chengdu, China; ^4^ Key Laboratory of Chronobiology (Sichuan University), National Health Commission of China, Chengdu, China; ^5^ The Joint Laboratory for Lung Development and Related Diseases of West China Second University Hospital, Sichuan University and School of Life Sciences of Fudan University, West China Institute of Women and Children’s Health, West China Second University Hospital, Sichuan University, Chengdu, China; ^6^ Department of Pediatric Pulmonology and Immunology, West China Second University Hospital, Sichuan University, Chengdu, China

**Keywords:** myeloid-derived suppressor cells, pulmonary hypertension, immune microenvironment, cancer-like, cytokines, chemokines, metabolism

## Abstract

Pulmonary hypertension (PH) is a chronic pulmonary vascular disorder characterized by an increase in pulmonary vascular resistance and pulmonary arterial pressure. The detailed molecular mechanisms remain unclear. In recent decades, increasing evidence shows that altered immune microenvironment, comprised of immune cells, mesenchymal cells, extra-cellular matrix and signaling molecules, might induce the development of PH. Myeloid-derived suppressor cells (MDSCs) have been proposed over 30 years, and the functional importance of MDSCs in the immune system is appreciated recently. MDSCs are a heterogeneous group of cells that expand during cancer, chronic inflammation and infection, which have a remarkable ability to suppress T-cell responses and may exacerbate the development of diseases. Thus, targeting MDSCs has become a novel strategy to overcome immune evasion, especially in tumor immunotherapy. Nowadays, severe PH is accepted as a cancer-like disease, and MDSCs are closely related to the development and prognosis of PH. Here, we review the relationship between MDSCs and PH with respect to immune cells, cytokines, chemokines and metabolism, hoping that the key therapeutic targets of MDSCs can be identified in the treatment of PH, especially in severe PH.

## Introduction

Pulmonary hypertension (PH) is a rare but severe vascular disorder, with a mean pulmonary arterial pressure (mPAP) ≥ 20 mmHg at rest (1). Pathologically, PH is characterized by pulmonary vascular remodeling (PVR) (2, 3). PVR involves proliferation and endothelial-mesenchymal transition of endothelial cells (ECs), phenotypic transformation and proliferation of pulmonary arterial smooth muscle cells (PASMCs), and proliferation, migration and excellular cell matrix (ECM) deposition of adventitial fibroblasts (AFs), as well as infiltration of inflammatory cells (4–8). In clinical practice, PH is classified into 5 groups based on etiology, pathophysiology and treatment, including group 1 [pulmonary arterial hypertension (PAH)], group 2 (PH associated with left heart disease), group 3 (PH associated with lung diseases and/or hypoxia), group 4 (PH associated with pulmonary artery obstructions) and group 5 (PH with unclear and/or multifactorial mechanisms) (1). Treatment of PH typically begins with primary therapy aimed at the underlying causes, and patients always die due to right heart failure. For nearly two decades, novel therapeutic agents, such as sotatercept and riociguat, have made progressions in the treatment of patients with PH (9, 10). However, the drugs only target to pulmonary vascular tone, and it is still difficult to reverse PVR. Therefore, to explore a new effective therapy for PVR is urgent. Recently, increasing evidence shows that immune microenvironment in adventitia of pulmonary arteriole plays an important role in PH development (11, 12). In healthy organisms, the immune system could balance the persistent equilibrium state between injury and repair by many sophisticated mechanisms, and distinct immunosuppressive cells could protect against excessive tissue damages (13). However, in chronic diseases like tumors, chronic infections and autoimmune diseases, inflammatory cells may establish a strong immunosuppressive microenvironment that suppresses anti-tumor and inflammatory immune responses, and promotes disease progression (14–16). Myeloid-derived suppressor cells (MDSCs) are a group of immunosuppressive cells that play an important role in PH development (17), and MDSCs may serve as a new therapeutic target for reversing PVR. Therefore, this review focuses on the relationship between PH and MDSCs, including cancer-like proliferation and immune microenvironment in PH, the phenotype of MDSCs, and the links between MDSCs and PH.

### Immune microenvironment and cancer-like process in severe PH

Severe PH is an incurable disease. Plexiform lesion, also called angiomatous malignant proliferation, is a morphologic hallmark of severe PH (18–20), which is a complex and disorganized pulmonary arterial proliferative lesion, consisted of vascular channels lined by a continuously proliferating endothelium and separated by core cells. The core cells could be myofibroblasts, PASMCs, ECs and undifferentiated cells (21). In 2008, Rai et al. (22) formally introduced the concept of cancer-like features in severe PH. Until now, accumulating evidence have supported that ECs, PASMCs and AFs involved in PVR demonstrate a hyperproliferative and apopotosis-resistant phenotype, and those cells are characterized by abnormally cancer-like growth characteristics (23–26). The cancer-like proliferative characteristics of vascular cells lead to irreversible changes in severe PH, but on the plus side, they also provide a new framework for antiproliferative and antiangiogenic therapy in severe PH. For the treatment of PH, especially severe PH, as Cool et al. (27) suggest, future treatment strategies will target immune mechanisms. If we want to re-open occluded pulmonary arterioles or halt disease progression, we might get some inspiration from cancer research data and concepts. In recent years, tumor immunotherapy has been effectively applied in clinical practice by the virtue of its targeting specificity (28, 29). Investigations of immune microenvironment and new targets for immunotherapy have become a hot topic in the field of tumor treatment, thus leading a new direction for exploring the targets of cancer-like PH.

**Figure 1:**
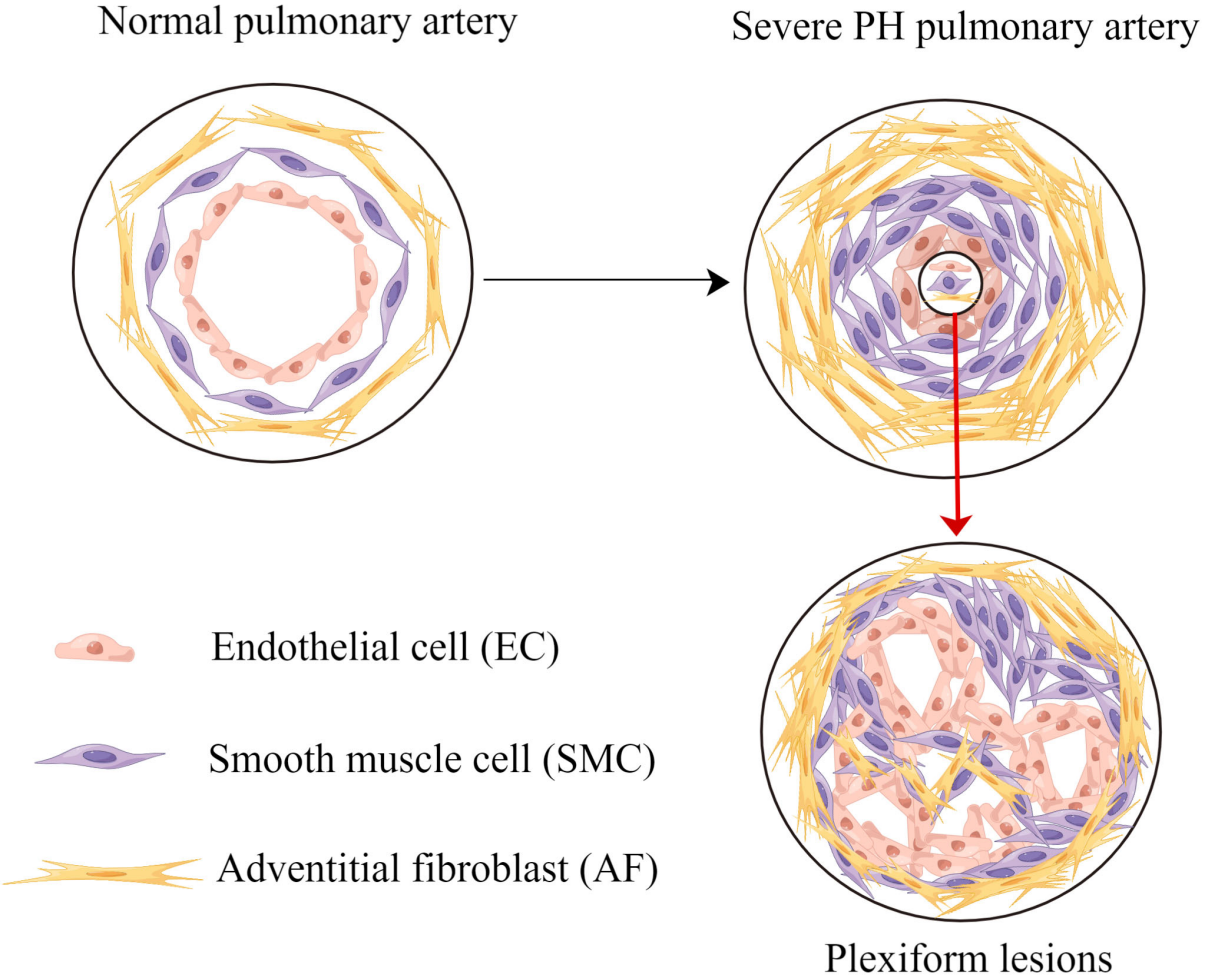
Pulmonary vascular remodeling and cancer-like growth. Pulmonary vascular remodeling causes endothelial cells apoptosis resistance, smooth muscle cells proliferation and adventitial fibroblasts proliferation. Without effective intervention, these processes lead to plexiform lesions and severe PH. PH, pulmonary hypertension.

Pulmonary vascular remodeling and cancer-like growth are summarized in **Figure 1**.

Pathological histology in patients and animal models of PH shows various degrees of inflammatory infiltration around the pulmonary vasculature. Inflammatory cells like T cells, macrophages, dendritic cells (DCs) and natural killer (NK) cells are observed around small pulmonary arteries. Abnormally high levels of circulating cytokines and chemokines are detected in peripheral blood (11, 30–32). In the past, most studies focus on macrophages, neutrophils, and DCs, to explore inflammatory microenvironment (33–35). Nowadays, more and more studies suggest that MDSCs could also play an important role in inflammatory microenvironment (17, 36–38). Under physiological conditions, MDSCs are important immune cells for hematopoietic stem cells to maintain homeostasis (39). Under pathological conditions, recruitment of MDSCs from circulating blood to the outer membrane of pulmonary artery may be an important mechanism for PVR and PH progression (17, 38). Thus, there is an imperative need to explore MDSCs in PH, and targeting MDSCs may provide clinical guidance for the treatment of PH.

### Myeloid-derived suppressor cells

MDSCs are a heterogeneous population of immature cells derived from myeloid progenitors with potent immunosuppressive activity (40, 41). In healthy mice, MDSCs constitute 20-30% of normal bone marrow (BM) cells and 2-4% of all nucleated splenocytes (42, 43). In normal myelopoiesis, immature myeloid cells differentiate into neutrophils and monocytes (44, 45). However, in pathological and chronic inflammatory conditions, such as tumors, chronic infections, and autoimmune diseases, a block in the normal myeloid differentiation occurs, and the persistent stimulation of myelopoiesis results in the expansion of MDSCs (41, 46). Thus, MDSCs in the BM are increased and released into the peripheral blood, and then migrate into various tissues of the body, especially around the lesions, to exert immunosuppressive functions.

Although MDSCs have been recognized for almost 30 years (47), the functional importance of MDSCs in the immune system is appreciated recently. MDSCs have been shown to be a potentially promising and well-tolerated therapeutic approach in tumor (48–50). The role of MDSCs in the PH process has not been fully illustrated yet. Elucidation of the immunosuppressive function and manipulation of the MDSCs phenotypes are therefore essential to fully understand.

### Phenotype of MDSCs

There are two subgroups of MDSCs according to their cell origins: polymorphonuclear MDSCs (PMN-MDSCs) and monocytic MDSCs (M-MDSCs). In human, CD11b and CD33 are used as pan markers for MDSCs (41). However, CD11b and CD33 are expressed not only in PMN-MDSCs and M-MDSCs but also in neutrophils and monocytes, so further studies on surface markers are required to distinguish specific cell populations of MDSCs (51, 52). The accepted markers of PMN-MDSCs in human are CD33^+^CD11b^+^CD14^-^CD15^+^/CD66b^+^ (41). In 2016, Condamine reported that LOX-1 was practically undetectable in neutrophils in the peripheral blood of healthy donors, whereas 5-15% of total neutrophils in patients with cancer and 15-50% of neutrophils in tumor tissues were LOX-1(+) (53). LOX-1 is a class E scavenger receptor expressed on macrophages, chondrocytes, ECs and smooth muscle cells (54). LOX-1(+) neutrophils have potent immunosuppressive activity, up-regulation of ER stress and other biochemical characteristics of PMN-MDSCs, so LOX-1(+) is proposed as a specific marker for human PMN-MDSCs (53). CD13^hi^ neutrophil-like MDSCs are found to be more immunosuppressive-active than CD13^lo^ neutrophil-like MDSCs in human pancreatic cancer, and CD13^hi^ is associated with poor prognosis, suggesting CD13^hi^ may be a new marker for PMN-MDSCs (55). Human M-MDSCs are described as CD33^+^CD11b^+^CD14^+^CD15^-^HLA^-^DR^-/lo^ (41). Monocytes, macrophages and DCs are mononuclear phagocytes that express major histocompatibility complex (MHC) class II molecules, whereas M-MDSCs usually lack MHC class II (56, 57). S100A9 is a new marker for monocytic human MDSCs (58). In humans, CD84 is expressed in both M-MDSCs and PMN-MDSCs, and CD84 has been identified as a marker of MDSCs in human cancer (40)

In mice, CD11b^+^ and Gr-1^+^ are used as pan markers, and the marker of PMN-MDSCs is CD11b^+^Ly6G^+^Ly6C^low^, whereas that of M-MDSCs is CD11b^+^Ly6G^-^Ly6C^hi^ (40, 41). In models of inflammatory bowel diseases and tumor-bearing mice, the CD49d^+^ subset of MDSCs is mainly monocytic and strongly suppress proliferation of antigen-specific T cells *via* a nitric oxide-dependent mechanism, which are similar to Gr-1(dull/int.) MDSCs. Thus, CD49d may potentially be a new marker to replace Gr-1 (59). Secreted protein acidic and rich in cysteine (SPARC) is a matrix protein, which can specifically control MDSCs suppressive activity, and SPARC is proposed as a new potential marker of MDSCs (60). CD84^hi^ MDSCs exhibit T cell-suppressive capacity and increased reactive oxygen species (ROS) production, thus CD84 has also been identified as a marker of MDSCs in cancer of mice (40).

The human and mouse MDSCs phenotypes are presented in **Table 1**.

**Table 1:** Human and mouse phenotypes of MDSCs.

Species	Standard phenotypical markers of MDSCs	Standard phenotypical markers of PMN-MDSCs	Standard phenotypical markers of M-MDSCs	Novel markers of PMN-MDSCs	Novel markers of M-MDSCs
Human	CD11b^+^CD33^+^	CD33^+^CD11b^+^CD14^-^CD15^+^/CD66b^+^	CD33^+^CD11b^+^CD14^+^CD15^-^HLA^-^DR^-/lo^	CD15^+^/CD66b^+^ CD14^-^LOX1^+^; CD15^+^/CD66b^+^ CD14^-^ CD84^+^; CD13^hi^CD11b^+^ CD33^+^ CD14^-^CD15^+^	CD14^+^/CD66b^-^CD84^+^ S100A9
Mouse	CD11b^+^Gr-1^+^	CD11b^+^Ly6G^+^Ly6C^low^	CD11b^+^Ly6G^-^Ly6C^hi^	CD11b^+^ Ly6G^+^ CD84^+^	CD11b^+^Ly6G^-^Ly6C^hi^CD84^+^

In rats, CD11b/c and His48 are used as pan-MDSC markers (61). Literature on rat MDSCs is limited and MDSCs are not uniformly labeled. We find that approximately 13 papers report MDSCs in rats by searching PubMed and Web of Science (61–73). CD11b/c^+^ and His48^+^ cells were first used as markers of MDSCs in T9 glioma rats in 2002 (61). Among the 13 studies, four use CD11b/c^+^ and His48^+^ as markers of MDSCs (61, 63, 64, 67), and one use CD11b/c^int^ and His48^hi^ (69). However, CD11b/c and His48 are expressed not only on MDSCs, but on neutrophils, monocytes, macrophages and DCs. Some studies use CD172a as a myeloid cell marker instead of CD11b/c (68), and some use CD161 as an alternative indicator of His48 in MDSCs (71). The phenotypes of rat MDSCs are summarized in **Table 2**. The studies on MDSCs in rat models are limited, and there are almost no specific markers to distinguish PMN-MDSCs and M-MDSCs. Therefore, further studies are needed to identify the cell markers of PMN-MDSCs and M-MDSCs in rats.

**Table 2:** Rat phenotypes of MDSCs.

Study Autor(ref.), time	Makers of MDSCs	Test specimen	Reference
Prins RM, 2002	CD11b/c^+^ His48^+^	spleen, glioma	(61)
Dugast AS, 2008	CD3^-^CD11b^+^CD80/86^+^	blood	(62)
Jia WT, 2010	CD11b/c^+^His48^+^	glioma	(63)
Zhang C, 2012	CD11b/c^+^His48^+^	bronchoalveolar lavage fluids	(64)
Dilek N, 2012	CD3^-^MHC class II^-^CD80/86^+^	blood	(65)
Lu YQ, 2013	CD11b/c^+^Gra^+^	blood, bone-marrow, spleen	(66)
Lin wy, 2014	CD11b/c^+^ His48^hi^	blood	(67)
Dolen Y, 2015	CD172a^+^His48^+^ Rp-1^-/+^	blood, spleen	(68)
Huaux F, 2016	CD11b/c^int^ His48^hi^	peritoneal lavage fluids	(69)
Azuma H, 2017	CD11b/c^+^MHC class II^-^	spleen	(70)
Hamdani S, 2017	CD11b/c^+^CD161^int^MCH class II^-^	spleen	(71)
Zhang FT, 2020	CD11b^+^Gr-1^+^	blood, spleen, bone-marrow	(72)
Liu JY 2021	CD11b^+^ His48^+^	blood	(73)

### Immunomodulatory functions of MDSCs in PH

The presence of MDSCs is confirmed in clinical patient samples and mouse models of PH (17, 36). MDSCs are significantly increased and positively correlated with mean pulmonary artery pressure in the PH patients group compared to the control group (36). In addition, pulmonary vascular muscularization, right ventricular remodeling and the worsening of PH are associated with the increase in pulmonary MDSCs, particularly PMN-MDSC (37). PMN-MDSCs mediate immunosuppressive effects through upregulation of arginase 1(Arg 1), ROS and prostaglandin E2, whereas M-MDSCs mediate the capacity through increasing expression of nitric oxide (NO), immunosuppressive cytokines including interleukin-10 (IL-10) and transforming growth factor-β (TGF-β), and immune regulatory molecules like programmed death-ligand 1 (PD-L1) (40). Increased levels of Arg 1, ROS, inducible nitric oxide synthase (iNOS) and TGF-β exacerbate the progression of PH (74–78). Group 3 PH is associated with lung diseases and/or hypoxia, and it is found the MDSCs are dominated by PMN-MDSCs, which exert the immune suppressive function through Arg1 and ROS (36, 37, 40). It is suggested that MDSCs are important constituents of immune microenvironment with a pivotal role in PH progression (38). Next, we focus on the chemokines, cytokines, T cells, NK cells and molecular mechanisms to explore the immune-regulatory functions of MDSCs in PH, which may provide us with new strategy for treatment of PH.

### MDSCs recruitment and activation: Cytokines and chemokines

**Figure 2:**
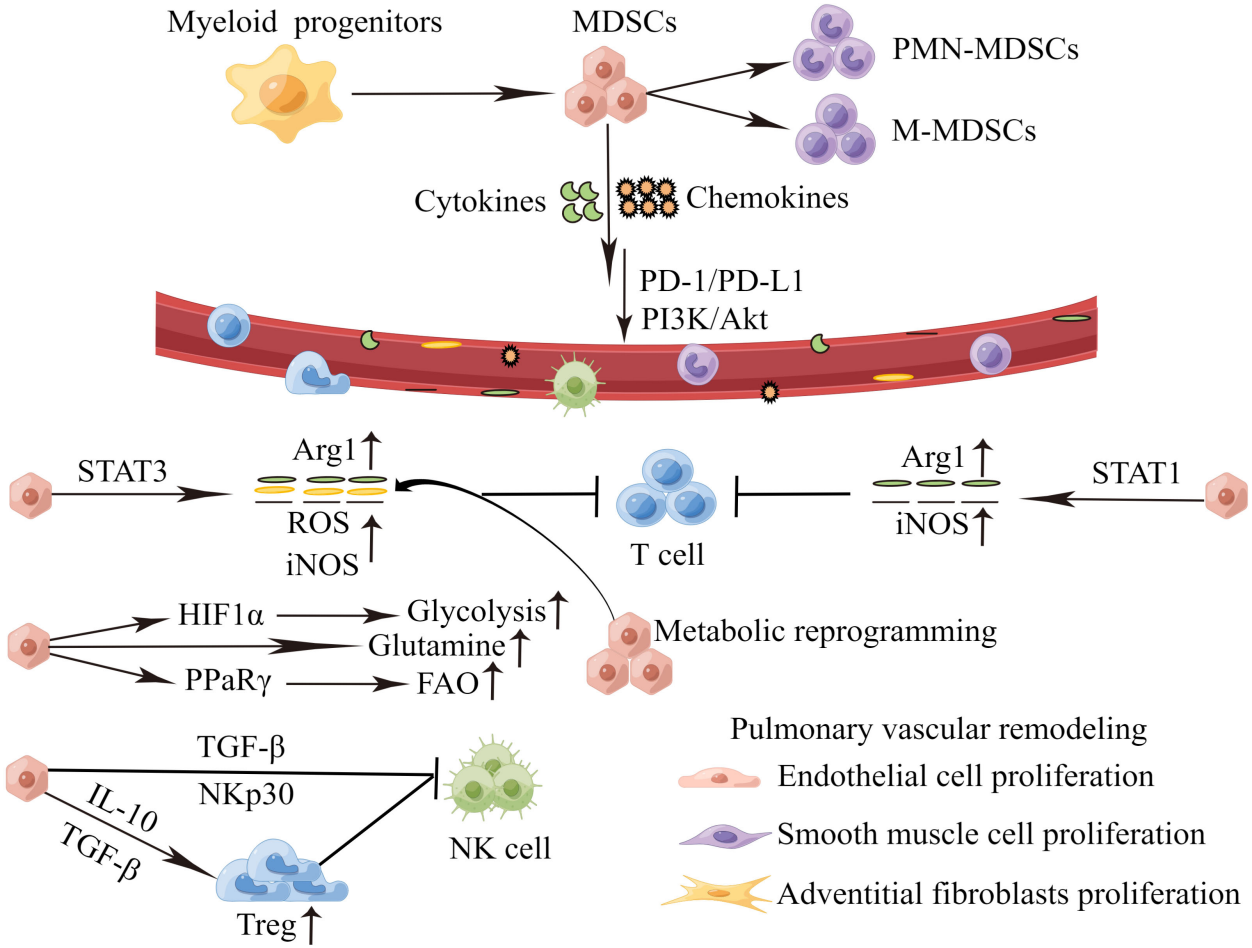
MDSCs recruitment and activation during PH progression. Perivascular immune cells constantly release inflammatory mediators, and the persistent stimulation of myelopoiesis results in the expansion of MDSCs in the BM. The MDSCs migrate to the pulmonary vascular site through the interaction between CCR,CXCR and respective chemokines (CCL,CXCL). In the PH microenvironment, MDSCs are activated and strongly inhibit an anti-inflammation reactivity of T cells and NK cells *via* various mechanisms. which in turn cause proliferation of endothelial cells, smooth muscle cells and adventitial fibroblasts, inducing pulmonary vascular remodeling. MDSCs, myeloid-derived suppressor cells; PMN-MDSCs, polymorphonuclear MDSCs; M-MDSCs, monocytic MDSCs; PD-1/PD-L1, programmed death 1 and programmed death-ligand 1; PI3K-Akt, phosphatidylinositol 3-kinase/Akt. Arg1, arginase 1; ROS, reactive oxygen species; iNOS, inducible nitric oxide synthase; HIF1α, hypoxia-inducible factor 1α; PPaRγ, peroxisome proliferator-activated receptor γ; FAO, fatty acid oxidation; TGF-β, transforming growth factor*-*β; IL-10, interleukin-10; NK cell, natural killer cells.

Chemokines and cytokines play a pivotal role on MDSCs recruitment and activation during PH progression, which are summarized in **Figure 2**. High levels of CXCL12 and CXCR4 are found in the lung tissues of PH patients, and PI3K/Akt signaling mediates CXCL12/CXCR4 regulation of proliferation and cell cycle progression in PASMCs, thus leading to PVR (79). Concurrently, the CCL2/CCR2 and CCL5/CCR5 pathways are necessary for cooperation between macrophages and PASMCs to initiate and expand PASMCs in migration and proliferation during PH development (80, 81). MDSCs express a wide range of chemokine receptors, including CCR2, CXCR2 and CXCR4 (82). In PH, the migration of MDSCs from BM to lesions may mediate by chemokines receptor/chemokine signaling, such as CXCL12/CXCR4, CXCL2/CXCR2 and CCL2/CCR2. In PH mice models, Bryant et al. find that PMN-MDSCs are transported to the lung through the chemokine receptor CXCR2, and promote the disease development (17). Deletion of CXCR2 in myeloid cells attenuates the recruitment of PMN-MDSCs to the lung microenvironment, and therefore inhibits PVR, and protects against PH (17, 83). In tumors, CXCR4 overexpression promotes infiltration of MDSCs in lung tissues. It will accelerate lung cancer progression and promote lung metastasis from other primary tumors (84–86). Recently, it has been suggested that targeting pulmonary tumor microenvironment with CXCR4-inhibiting nanocomplex enhances anti-PD-L1 immunotherapy (84). Cytokines are significantly increased in patients with PH. IL-6, IL-1β and IL-18 can induce the proliferation, migration, and differentiation of pulmonary vascular cells, thereby promoting PVR (11, 87–89). Meanwhile, cytokines may promote the recruitment of MDSCs, exacerbate the inflammatory response in the blood vessels, and aggravate the disease. Thus, cytokines, chemokines and MDSCs contribute to the formation of the immune microenvironment of PH and play a key role in the pathogenesis of PH. Targeting cytokines and chemokines to inhibit MDSCs infiltration in PH is little studied. Fortunately, there are more and deeper studies in tumors. Targeting CXCR4 or CXCR2 can reduce MDSCs infiltration, which contributes to the inhibition of tumor growth and metastasis (90, 91). Targeting IL-6 or IL-6 receptors for tumor immunotherapy can block MDSCs-mediated immunosuppression (92). Therefore, targeting chemokines and cytokines might be a potential new method for reversing PVR in PH.

### Target cells of MDSCs: T cells and NK cells

The T-cell immune response has a protective effect in PH, and an intact T-cell immune system is important in protecting against pulmonary angioproliferation (93). Athymic nude rats treated with the vascular endothelial growth factor (VEGF) receptor blocker SU5416 developed severe PH and PVR under normoxic conditions, whereas normal rats may not develop severe PH under normoxic conditions (94). Similarly, the reduction and deficiency of CD8+ cytotoxic T cells exacerbate the risk of death in PH patients (95). Regulatory T cells (Tregs) are a naturally occurring subpopulation of T cells with immunosuppressive functions, and Tregs primarily maintain autoantigenic immune tolerance and effectively control the exacerbation of inflammatory responses (96–98). Normally, Tregs regulate immune cells by controlling IL-2 availability, inhibitory receptors, and secretion of cytokines such as IL-10 and TGF-β, which may maintain immune homeostasis and suppress autoimmune damage (99). Tregs are dysfunctional in human patients and animal models of PH and may contribute to the development and progression of the disease (100). In chronic inflammatory diseases of the lungs, MDSCs can attenuate the role of helper T cells and cytotoxic T cells and exacerbate inflammatory cell infiltration around the pulmonary vasculature (101). MDSCs can likewise induce Tregs amplification, but the amplified Tregs exert a negative regulation of immunity (102), which promotes immune escape in tumors and autoimmune diseases. These factors may contribute to the possibility that MDSCs promote PH development.

NK cells are cytotoxic lymphocytes that are critical to the innate immune system and can recognize and rapidly kill target cells (103, 104). Emerging evidence indicates that MDSCs can interact with NK cells and regulate their functions (105). MDSCs can strongly inhibit anti-tumor immune responses of NK cells and promote the progression of tumors (106, 107). MDSCs suppress NK cells activation by inducing Tregs (108). In a mouse model of orthotopic liver cancer-bearing, it is shown that downregulation of NK cells function is inversely correlated with the marked increase in MDSCs in the liver and spleen, and MDSCs inhibit cytotoxicity, NKG2D expression and IFN-γ production of NK cells through membrane-bound TGF-β (109). Alternatively, co*-*culture of MDSCs and NK cells from hepatocellular carcinoma patients reveals that MDSCs-mediated inhibition of NK cell function is dependent mainly on the NKp30 on NK cells (110). MDSCs have an inhibitory effect on NK cells, mainly promoted by TGF-β or Tregs. NK cells impairment is a feature of PH and contributes to PVR in animal models of the disease (111). A prospective survival study of PH patients has confirmed a positive correlation between the number of NK cells and short-term survival in PH patients, suggesting that deficiencies in NK cells might be associated with an increased risk of death in PH patients (95). However, the cell interaction of MDSCs, NK cells and its molecular mechanisms have not been reported in PH yet, which needs further researches.

### Molecular mechanisms of MDSCs: Arginine, iNOS and metabolic mechanisms

It is well known that MDSCs regulate immune response through a variety of mechanisms, such as arginase, iNOS activation and energy metabolic dysregulation (112). MDSCs highly express the immunosuppressive molecule Arg 1. L-Arg depletion by MDSCs blocked the re-expression of CD3zeta in stimulated T cells and inhibited antigen-specific proliferation of OT-1CD8+ and OT-2 CD4+ T cells, which will impair T-cell functions and affect T-cell-mediated immune responses (113). MDSCs are detected in the peripheral circulation of patients with PH, and active MDSCs expression increases transcripts for Arg 1 (36). Arginase activity and alterations in arginine metabolic pathways have been implicated in the pathophysiology of PH (114). Arginase activity is elevated almost two-fold (p=0.07) in patients with PH (74). Dysregulation of arginine metabolism contributes to endothelial dysfunction and PH in sickle cell disease and is strongly associated with prospective patient mortality (115). Arginase inhibitors can reduce PVR and collagen deposition, and then prevent bleomycin-induced neonatal PH in rats, and may prevent inflammation and remodeling in a guinea pig model of chronic obstructive pulmonary disease (116, 117). Arginase catalyzes the degradation of L-arginine to L-ornithine and urea. The decrease of L-arginine will impair T-cell functions and decrease NO production, and may exacerbate PVR and accelerate PH development.

Three distinct genes encode three NOS isoforms: neuronal nitric oxide synthase (nNOS), endothelial nitric oxide synthase (eNOS), and iNOS (118, 119). MDSCs exerted their inhibitory function on T cells in an iNOS-dependent manner (120). iNOS is an enzyme that catalyzes the formation of NO and citrulline from L-arginine, which can lead to the block of T-cell synthesis by reducing L-arginine, and can also inhibit the functions of T cells by producing excessive NO (121, 122). In hypoxic PH, the iNOS is found to be upregulated and involved in the formation of PH (123, 124). Mice lacking iNOS are protected against emphysema and PH (125). In a smoke-exposed mice study, iNOS expression in BM-derived cells drives pulmonary vascular remodeling (125). iNOS deletion in myeloid cells confers protection against PH and provides evidence for an iNOS-dependent communication between M2-like macrophages and PASMCs in underlying pulmonary vascular remodeling (126). Treatment of wild-type mice with the iNOS inhibitor N (6)- (1-iminoethyl)-L-lysine (L-NIL) prevents structural and functional alterations in the lung vasculature and alveoli and reverses emphysema and PH (127). In hypoxia-induced PH, iNOS may release large amounts of NO and damage the vascular endothelium, and the endothelial damage will diminish NO bioavailability (75, 128, 129). MDSCs can promote immune suppression by the production of ROS (40). In PH, the overproduction of ROS contributes to pulmonary vasoconstriction, muscularization of pulmonary arterioles, perivascular fibrosis and PVR (130). MDSCs highly express iNOS and produce ROS that may exacerbate PVR through either a direct or an indirect mechanism.

Metabolic dysregulation has emerged as a major area of research in the pathobiology of PH (131). Compared to that of healthy individuals, the microenvironment of PH has different metabolic features, such as excessive intracellular glucose uptake, increased glycolytic metabolism, insulin resistance, and alterations in high-density lipoprotein (HDL), cholesterol and leptin (24, 132). The metabolism of MDSCs, such as glycolysis, fatty acid oxidation and amino acid metabolism, is reprogrammed in the tumor microenvironment (40). In tumor, the MDSCs metabolic reprogramming enhances the immunosuppressive activity of Arg1 and iNOS, which will lead to apoptosis of effector T cells and suppression of cell proliferation, and promote tumor proliferation and metastasis (133, 134). Under hypoxic conditions, the activation of hypoxia- inducible factor 1α (HIF-1α) induces the switch from oxidative phosphorylation to glycolysis in MDSCs (40). The activation of the HIF-1α pathway promotes the immune suppressive activity of MDSCs (135). Blocking lactate production in tumor cells or deleting HIF-1α in MDSCs reverse anti-tumor T-cell responses and effectively inhibite tumor progression after radiotherapy for pancreatic cancer patients (136). Lipid metabolism plays a key role in the differentiation and functions of MDSCs. MDSCs exhibit fatty acid uptake and increase fatty acid oxidation (FAO), which support the immunosuppressive functions of MDSCs (137). Tumor-infiltrating MDSCs in mice may prefer fatty acid oxidation (FAO) as a primary energy source, while treatment with FAO inhibitors improves anti-tumor immunity (138). Mouse and human PMN-MDSCs upregulate fatty acid transport protein 2 (FATP2), and the selective pharmacological inhibition of FATP2 abrogates the activity of PMN-MDSCs and substantially delays tumor progression (139). MDSCs can regulate T cells functions by depriving the essential metabolites, such as arginine, tryptophan and cysteine from the microenvironment (40). MDSCs can deplete arginine through upregulation of Arg1 and reduce tryptophan through upregulation of indoleamine 2,3-dioxygenase, which suppress T-cell proliferation and activation (140, 141). In a triple-negative breast cancer immunotherapy-resistant model, targeting glutamine metabolism also significantly inhibites the production and recruitment of MDSCs and suppresses tumor growth (142). Thus, MDSCs may also promote disease development by suppressing T-cell functions through metabolic pathways in PH.

## Conclusion

The complex changes in cytokines, chemokines, and immune cells in PH and their association with PVR suggest that immune mechanisms play an important role in PH. The cancer-like growth characteristic is one of the important features of severe PH. The prognosis of severe PH is even worse than most of the cancers in children. Hence, it is urgent to find a novel therapeutic strategy for the cancer-like PH. Tumor immunotherapy has been effectively applied in clinical practice by virtue of its specificity and targeting. Recently, there is ample evidence to demonstrate that tumor immunotherapy can be effectively improved by targeting MDSCs. Just as in tumors, MDSCs play a crucial role in development and progression of PH. Therefore, targeting MDSCs may be a potential protocol in the treatment of PH to arrest PVR, including blocking the migration, recruitment, activity and metabolism, and promoting the maturation of MDSCs to restore immune homeostasis. We believe that the MDSCs‐targeting treatment can provide a first-line survival opportunity for patients with PH, especially severe PH.

## Author contributions

Conceptualization HZ. Original draft preparation HZ, QL, YL, XT. Review LG, HL. All authors contributed to the article and approved the submitted version.
